# Inferring Biological Structures from Super-Resolution Single Molecule Images Using Generative Models

**DOI:** 10.1371/journal.pone.0036973

**Published:** 2012-05-22

**Authors:** Suvrajit Maji, Marcel P. Bruchez

**Affiliations:** 1 Lane Center for Computational Biology, School of Computer Science, Carnegie Mellon University, Pittsburgh, Pennsylvania, United States of America; 2 Department of Biological Sciences, Carnegie Mellon University, Pittsburgh, Pennsylvania, United States of America; 3 Department of Chemistry, Carnegie Mellon University, Pittsburgh, Pennsylvania, United States of America; Uni. of South Florida, United States of America

## Abstract

Localization-based super resolution imaging is presently limited by sampling requirements for dynamic measurements of biological structures. Generating an image requires serial acquisition of individual molecular positions at sufficient density to define a biological structure, increasing the acquisition time. Efficient analysis of biological structures from sparse localization data could substantially improve the dynamic imaging capabilities of these methods. Using a feature extraction technique called the Hough Transform simple biological structures are identified from both simulated and real localization data. We demonstrate that these generative models can efficiently infer biological structures in the data from far fewer localizations than are required for complete spatial sampling. Analysis at partial data densities revealed efficient recovery of clathrin vesicle size distributions and microtubule orientation angles with as little as 10% of the localization data. This approach significantly increases the temporal resolution for dynamic imaging and provides quantitatively useful biological information.

## Introduction

Super-resolution (SR) imaging has recently led to a number of important insights in biology that could not have been achieved with conventional microscopy due to optical resolution limitations [1], [2]. A variety of approaches now achieve resolution far beyond the diffraction limit. Localization based approaches such as STORM [3], PALM [4], FPALM [5] and related methods have been employed effectively for static and slowly-moving structures. These approaches require sequential acquisition of positions of individually resolved fluorescent molecules, which are then assembled into a high-resolution image. The resolution in these images is related to the localization accuracy and the sampling density, with high-resolution images requiring comprehensive sampling of the molecular positions. Because of these requirements, localization microscopies still struggle to provide high spatial and temporal resolution images, primarily due to the time-scale mismatch between acquisition and biological motion. Recent demonstrations using very high laser power improved the frame-capture timescale by an order-of-magnitude by accelerating the localization and deactivation cycle time [6]. While this approach achieved 0.5–2 second acquisition speeds, this still poses a challenging limit for many biological processes.

Recently, computational methods from the branch of statistical machine learning and computer vision [7]–[12] have been applied to biological structures and biophysical processes. Various generative models [13]–[15] have been used to facilitate analysis of conventional microscopy images. The structure of localization-based SR imaging data is different than that of conventional microscopy. The catalog of molecular positions provided by this approach provides information about the underlying structures at molecular length scales. Such data requires computational approaches that utilize the inherent positional information to extract meaningful structural biology–scale information about those cellular structures. Because localization microscopy relies on sequential acquisition of molecular positions, a shorter acquisition window results in identification of fewer molecular positions from the underlying structure. Dynamic localization datasets are inherently incomplete; yet represent a statistical sampling of the complete underlying structure. We hypothesized that generative models can accurately identify underlying biological structures at high resolution using significantly less data. Such models can be used to extract useful biological information such as characteristic lengths and inclination angles of filamentous structures, organelle size and shape and other representative characteristics of the underlying structures.

Here we apply a parametric feature extraction method known as the Hough Transform [16] to identify basic structures using sparse single molecule (SM) data in 2-d. This approach is robust to noise sources common in localization datasets. In addition, it is robust to occlusion and the presence of features unrelated to the parameterized features of interest. As implemented here, the Hough Transform efficiently infers underlying structures in spite of substantially reduced molecular sampling density and recovers quantitatively useful information about the sample set based on the parametric definitions of the objects. This computational framework lays the groundwork for extension to more generalized parametric objects in 2-d and 3-d.

The Hough Transform (HT) and its close relative Radon Transform has been previously used to study biological features from images [8], [17]–[19]. We extend the method to the analysis of localization based super resolution image datasets. Although we evaluate only the parametric case, the generalized Hough Transform (GHT) and variants can be extended to non-parametric cases. In case of the standard HT applied here, the parameter space for lines is 2-d and for circles is 3-d, both remaining computationally tractable for typical SR datasets [16]. In contrast, GHT variants usually involve a 4-d parameter space with position, orientation and scale [20], and are substantially more computationally expensive. An efficient extension of GHT called displacement vector GHT (DV-GHT) is proposed in [21]. Some other improved and faster variants have been proposed for 2-d [22]–[29] and 3-d [30]. HT and GHT are inherently parallelizable, so large-scale computation can be managed by performing hardware-based parallel processing using the latest GPUs [31] or field programmable gating arrays (FPGA) [32], potentially making some of these generalized methods computationally approachable.

## Results

### Simulated Data Generation

**Figure 1:**
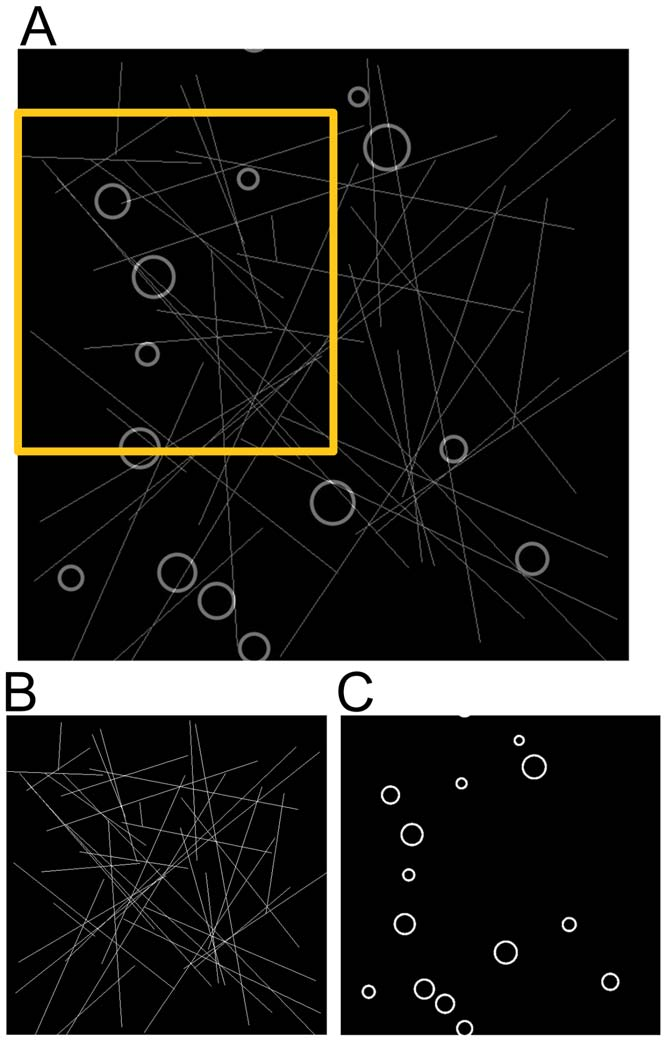
Structural mask for simulated data. (A) Lines and Circles, cropped image in the yellow rectangle box is shown in Figure 2. (B) lines only (C) circles only.

The basic structural elements in biology are often simple geometric shapes such as lines, circles and ellipsoids [33]. To mimic filamentous structures such as actin fibers or microtubules and circular shaped structures such as clathrin-coated pits or endosomes we have generated artificial data consisting of binary lines and circles in distinct channels (Fig. 1). The density of lines in the example mask corresponds to real biological structures such as lamellipodial actin networks [34] if the mask area represents a 640 nm×640 nm region of a cell (a 1 pixel = 1 nm^2^ scale). Active pixel points from the mask structures are randomly selected to simulate stochastic activation of fluorescent molecules, analogous to PALM and STORM imaging. This reduces the selection bias of molecules from a certain region of the structures and retains the relative density of the molecules for all regions. For all simulated and real datasets, the found or simulated molecular positions were the input to the HT calculations. A number of papers have reviewed robust approaches for identifying molecular positions from localization datasets [35], [36].

### Noise Sources

The two basic noise sources in localization-based SR imaging are position noise (localization accuracy) and outlier noise (background signal) [4], [37]. The position noise represents the limitations inherent in finding the true position of a molecular emitter, while the outlier noise represents spurious localizations and nonspecific fluorophore binding sites typical of real datasets. Outlier noise was generated as ‘Salt and Pepper’ noise in MATLAB although any type of noise can be considered. The position noise of 0, 5 and 10 pixels represent the FWHM of the Gaussian spread of position relative to the true active-pixel location in the mask. Outlier noise densities tested were 0, 0.002, 0.005, 0.01, 0.02 and 0.05 expressed as the fraction off-mask pixels considered as a found molecular position. Outlier noise densities above 0.002 are extremely high for single molecule datasets and unrealistic, but were included to assess the robustness of the reconstruction method to high degrees of noise. Additional simulations were performed at other intermediate position noises. While only three cases are shown here all are available (Fig. S2 and Movies S1, S2, S3, S4, S5, S6, S7).

**Figure 2:**
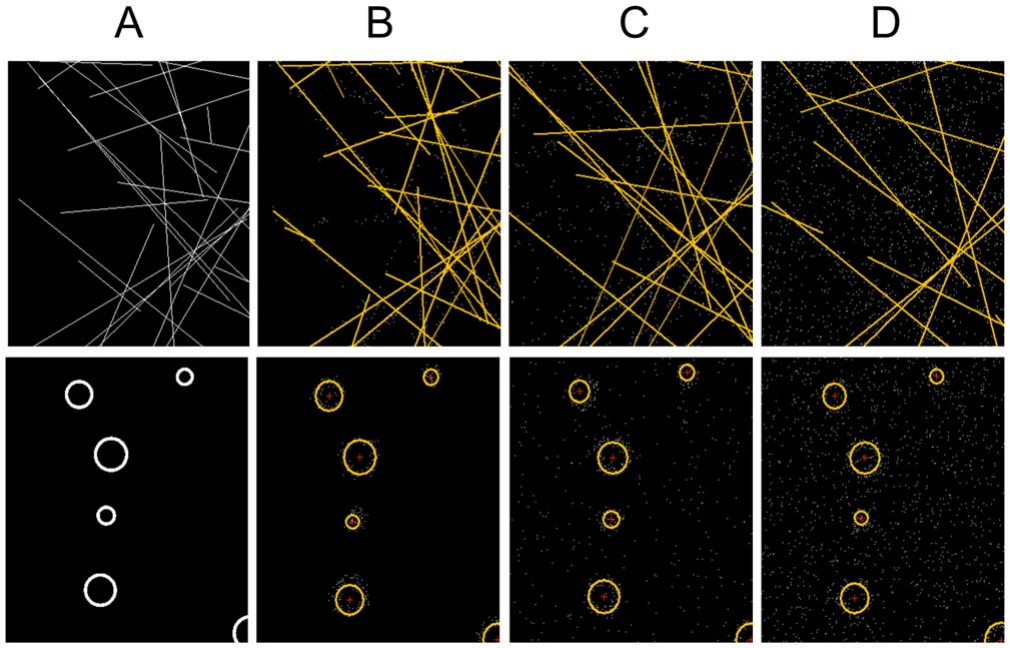
Representative linear and circular structure reconstruction. Column (A) Mask (B) outlier noise density 0 (C) outlier noise density 0.005 (D) outlier noise 0.02. Position noise is 5 pixels with data density of 15% for all cases here.

**Figure 3:**
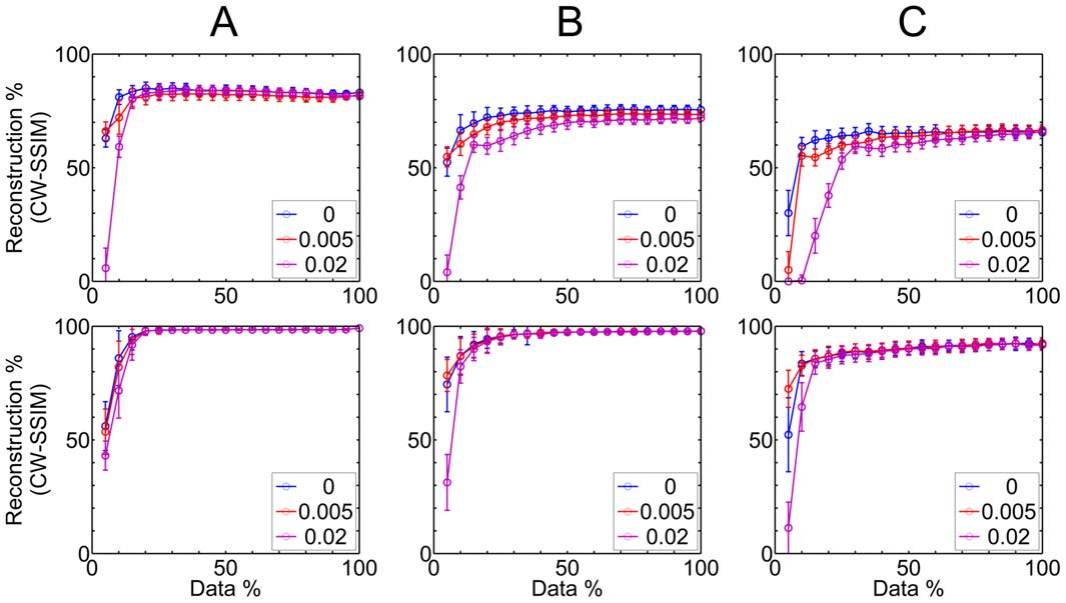
Reconstruction measure using Structural Similarity Index CW-SSIM. A total of 100 random simulations were performed at each data density and at outlier noise densities of 0, 0.005 and 0.02. Top row is for lines and bottom row is for circles. Column (A) Position noise of 0 pixel. (B) Position noise of 5 pixels. (C) Position noise of 10 pixels. Reconstruction measure for all the noise densities are shown in Figure S2.

Simulations of the linear and circular masks at different outlier noise, position noise and sampling density demonstrated that the HT is able to reconstruct the linear and circular structures robustly and accurately at high outlier noise levels and position noise levels similar to those seen in real single molecule localization data [3], [4], [38]. The reconstructed lines and circles are shown in Fig. 2 and the reconstruction performance, quantified using a complex wavelet structural similarity index measure (CW-SSIM) is shown in Fig. 3.

### Reconstruction from simulated data

Figure 2 shows a cropped section of the reconstructed lines (top row) and reconstructed circles (bottom row) overlaid on the point datasets for the mask shown in Fig. 1A at different outlier noise densities, a position noise of 5 pixels and a data density of 15% (fraction of total number of possible points that constitutes the structure). The full reconstruction for lines and circles at all the position noise and outlier densities are shown in the Movies S1, S2, S3, S4, S5, S6, S7

The plots shown in the top row of Fig. 3A, 3B, 3C for lines reveals that at lower position noise cases, the reconstruction measure is close for different outlier noise densities; although as expected it is better at low outlier noise. In general the reconstruction gets better with increased sample density but beyond a data density of 10–15% (low position noise) and 15–20% (high position noise), more data does not provide more information about the structure and the CW-SSIM measure reaches a plateau. This indicates that collection of SM-SR data has an optimum value for dynamic experiments. The plots shown in the bottom row Fig. 3A, 3B, 3C for circles reveal a similar trend at various position noise and outlier noise to that of line reconstruction. The reconstruction for circles is significantly better than the lines, an improvement expected due to the 3-d parametric space for circles.

The HT is more robust to outlier noise than to position noise in these simulations. This is likely a result of the Hough accumulator which scores votes for objects that are coincident with a feature and does not account explicitly for localization uncertainties (objects that are near to a feature). Improvements to the algorithm could incorporate localization uncertainty directly.

On the whole, Fig. 3 demonstrates that most of the structural information can be recovered with only a fraction of the single molecule data for analysis of lines and circles. In this example mask, for lines, about 15% data identifies 80–85% or more of the input structures while for circles around 10% of the data identifies more than 90–95% of the input structures. Sampling beyond these levels only modestly increased the information recovery. For lines and circles, inclusion of additional data density beyond these levels only resulted in modest additional feature identification (<10%). With an improved HT we would likely improve the performance in recovering the dense linear structures, for example using Monte Carlo optimization over parameter space or maximum likelihood shape reconstruction [39].

**Figure 4:**
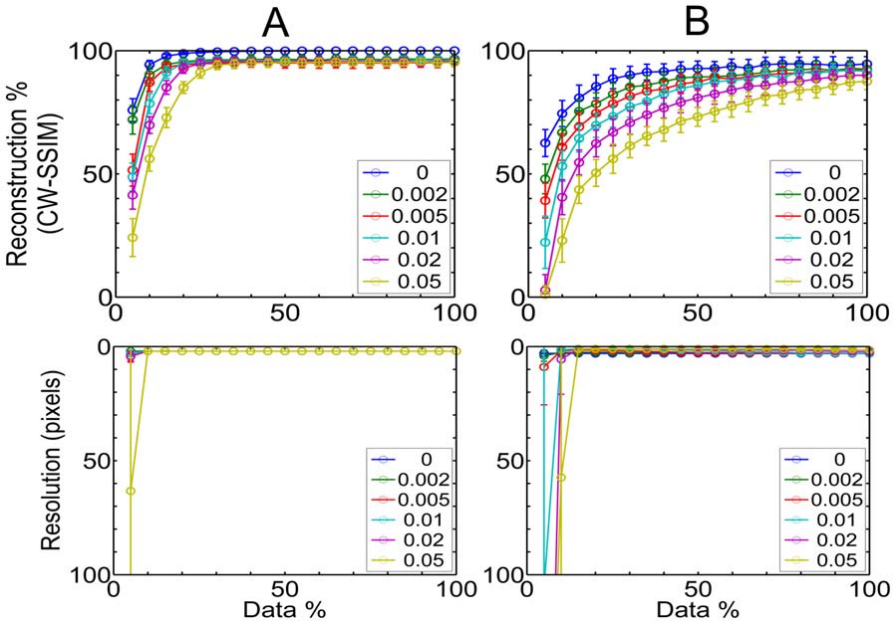
Parallel line reconstruction. Reconstruction measure using Structural Similarity Index CW-SSIM (top row) and resolution, calculated as the minimum inter line distance (bottom row) at indicated outlier noise densities. A total of 100 random simulations were performed at each data density. Column (A) Position noise of 0 pixel. Column (B) Position noise of 2 pixels.

We also performed similar analysis for parallel sets of lines to determine the resolution, calculated as the smallest pairwise distances between all the lines, at different data densities. The reconstruction result is shown in Figure 4 for the mask in Figure S4, and we found that the highest resolution is obtained at 10–15% of the input data. This is a marked contrast to the spatial sampling requirements according to the Nyquist theorem, requiring a measured molecular density at half the length scale of the smallest feature size in the data.

### Reconstruction from real data

**Figure 5:**
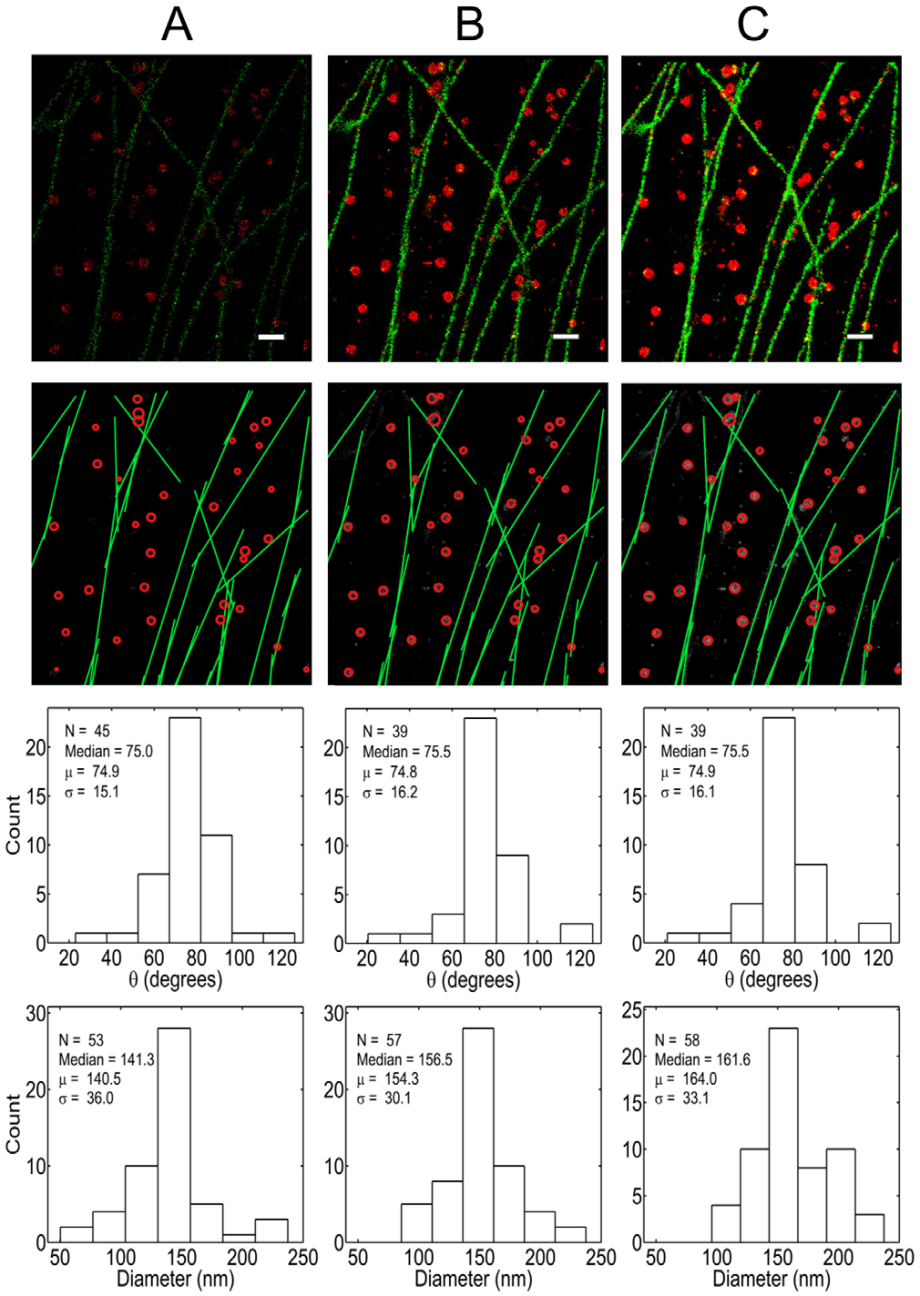
Single molecule localized data of clathrin (red) and tubulin (green). Top row is the plotted positions from both channels. Scale bar is 500 nm. Second row is the representative reconstructed structures from both channels, overlaid on the data (A) 10% data (B) 50% data. (C) 100% data. Third row is the histogram of orientation angle of the reconstructed line segments and the bottom row is the histogram of the diameters of the reconstructed circles.

We obtained the molecular position table from the previously published two-color STORM datasets [37] that labeled clathrin (red) and tubulin (green) in BS-C-1 cells. We applied the Hough Transform reconstruction for lines and circles independently on the two channels. The reconstruction is shown in Fig. 5 and the full reconstruction at more data densities is shown in Movie S8. It is not possible to determine the CW-SSIM without the actual structure, so the performance is gauged visually and with quantitative feature analysis. We have validated the robustness of the HT on the real data by performing the feature extraction and analysis with 100 random samplings at each of three data densities. The statistics from these analyses are shown in Table 1.The parameter extraction and distribution properties from the 100 random samplings are very consistent, evidenced by the negligible standard deviations in the mean and median parameter values. It should be noted that at 100% density the data remains the same for each sampling and hence the feature extraction is exactly the same for all the sampling instances with standard deviation of practically zero for all the parameter values. This method is robust to cross-talk (Fig. S3) (as explained in the methods section) of the multicolor channels and so it was not necessary to perform density filtering [37] prior to analysis.

**Table 1:** HT extracted feature parameter values for the real data over 100 random samplings at 10, 50 and 100% data density.

Data density		10%	50%	100%
*θ_average_*(degree)	*μ*	76.04	75.6	74.9
	*σ*	1.4	0.64	1.6×10^−13^
*θ_median_*(degree)	*μ*	75.8	76.4	75.5
	*σ*	1.02	1.1	0.0
D*_average_*(nm)	*μ*	137.2	152.8	164.0
	*σ*	3.5	1.5	2.6×10^−13^
D*_median_*(nm)	*μ*	138.1	156.5	161.6
	*σ*	6.4	2.6×10^−13^	2.6×10^−13^

*θ_average_* and *θ_median_* are the mean and median values of the orientation angle of all the lines for a particular sampling. D*_average_* and D*_median_* are the mean and median values of the diameter of all the circles. *μ* and *σ* are respectively the mean and standard deviation over the 100 random samplings for the average and the median values of the *θ* and *D* distributions.

The original data provided was in camera pixel coordinate space. We have performed the reconstruction at 25× scaling from the original coordinate space (∼6 nm×6 nm pixel-size). This scale retains most close points without being binned into the same pixel when we discretize the coordinates for analysis. Most of the structural information is obtained at just 10% of the single molecule localization data (Fig. 5) and very little additional information is recovered at higher data densities. This holds true for both the image reconstruction and the extracted distributions of quantitative traits from the objects. The quantitative information extracted from the HT parameters for objects identified in the tubulin and clathrin localization data is shown in the histograms of Fig. 5 (third row–tubulin, fourth row–clathrin). The histograms of tubulin orientation are practically identical with a mean and median of about 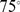 for the three data densities shown here. The distribution of clathrin vesicle diameters is also similar for the three data densities. The mean and median values of the distributions of clathrin diameters are slightly higher with increasing data density, increasing from 140 nm (10%) to 160 nm (100%), a likely consequence of the increased data density providing more votes from localizations at the periphery of the circular objects. As with any automated analysis, there are some missed structures and some spurious structures in the reconstruction. These represent ∼10% of the distinct features identified by manual inspection. The choice of parameters could be optimized iteratively to achieve the best possible solution.

We have compared this HT approach to an alternative feature extraction method. Blob detection [40] with the Laplacian of Gaussian (LoG) as the kernel is an established method for object detection generally applied to intensity images. We applied blob detection to datasets with 10% and 50% of the clathrin localizations included, and attempted to extract quantitative parameters from the blob analysis (Fig. S5). This approach generated multiple blob circles of different radii at multiple scales for the same feature, so we had to filter out the smaller circles with an aggressive size filter, eliminating some circles of a biologically relevant length scale. While this approach correctly locates the possible features, it tends to overestimate the circle size as can be seen from Fig. S5C and S5G and the diameter histograms S5D and S5H. Moreover since it does not discriminate between different feature types, it is not robust to cross talk from the other channel. For quantitative analysis of sparse localization data, the HT is significantly more robust than the blob detection.

## Discussion

Generative models allow efficient reconstruction of underlying parametric objects in both simulated and real localization microscopy datasets at data densities between 10–20%. These approaches substantially improve the efficiency of SM–SR imaging to generate quantitative biological and structural information. This approach can be potentially used with dynamic SM-SR imaging of structural components in cells to improve the temporal resolution by a factor of 5 to 10. Since the parameters of the method represent physical traits such as radius of circles or orientation angle of lines, we are able to extract meaningful and reliable distributions of object properties with this approach in both simulated and real datasets. More careful quantification of the parameter space could be used to extract, for example, the underlying molecular density for a feature, since the classical HT method is based on implicit Bayesian voting of the localized points in the datasets. It is also possible to obtain the persistence length of the tubulin from the obtained coordinates of the lines with further analysis.

The difference of estimated median clathrin vesicle diameter seen at different data densities in Fig. 5 is a result of the voting process. At lower data density the edge points are most likely underrepresented in the vote counts relative to high data densities. To overcome this issue we can apply weighted voting for circle detection so that even a small number of points towards the outer edge of the circles can receive enough votes to be considered as a valid shape. We have tested this correction, but found that the full normalization appeared to overestimate the boundary. The correct level of voting normalization could be estimated through a statistical learning of several such objects at low data density. Nevertheless, there is always systematic bias in estimating biological structures, from real biological experiments. In spite of this, quantitative comparisons across treatment conditions with similar data densities remain informative in assessing differences in biological datasets. The robustness of the HT-based feature estimation makes such an approach feasible.

As seen from the results section, the classical HT for line detection was limited to narrow filamentous structures since it has no accommodation for the uncertainty of the molecular position. Methods do exist for such purposes [41]. In this study we have shown that given sparse molecular positions we can generate the corresponding biological structures with high efficiency using simple shape primitives. Variants of the HT and other methods [42]–[47] can detect arbitrary shaped structures. Here we have applied only the classical form of the HT for inferring basic parameterizable biological shapes. This approach could be easily extended and improved by including parameter optimization through Monte Carlo sampling. Extension to arbitrary shapes could be accomplished using variants of the classical HT such as the Generalized HT [20], which can be used for shapes without a parametric form, Randomized or Probabilistic HT [24], or the Progressive Probabilistic HT [29]. These generative methods may be particularly useful for dynamic imaging of cellular components at high spatial and temporal resolution.

## Methods

### Hough Transform

The Hough Transform [16](HT) is a standard computer vision tool for recognition of global patterns in an image space by recognition of local patterns such as points or peaks in a transformed parameter space. The basic idea of HT method is to identify parametrizable curves such as lines, polynomials, circles, ellipsoids, and others using a voting procedure on the parameter space based on features in the image. Each input feature contributes to a global consensus shape that most likely generated the image point. Localization datasets produce discrete features, namely the set of found molecular positions. Since each point is treated independently, outlier noise pixels will add small peaks and occluded points will just alter the peak intensities in the parameter space without changing the actual structure. In addition, points from other shapes will not significantly contribute to the peaks for the consensus shape in the transformed parameter space. These traits make the HT robust to noise, partial occlusion and the presence of other shapes, common problems encountered with localization microscopy. HT does not require any prior information about the number of solution classes and can find multiple instances of the shape at once. We have applied the classical HT to extract linear and circular structures from SR biological datasets. HT implicitly generates the observable structural data from a probability density function through a Bayesian process [48]. Hence HT is an implicit generative model of parameterized shapes.

### Hough Transform as a Generative Model for Biological Structures Using Single Molecule Data

In classical machine learning a generative model is defined as a model that can randomly generate observable data with a parameter set defined by a full joint probability distribution with priors. The working principle of the Hough Transform (HT) is essentially a voting process. Investigated from a Bayesian perspective, if the votes follow a probability distribution, the joint probability distribution of all the input feature points is, in effect, the voting process. The mathematical proof has been shown elsewhere [48] for conventional images and edge points found through edge detection. In the current application, the features are localized single molecules from labeled biological structures that can be represented as parametric objects. The proof can be straight forwardly extended to this situation.

Parameterization of a structure is based on a function that defines the structure in terms of a set of variables. The parametric normal form of a line is:
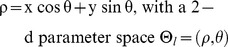
(1)The parametric equation for a circle is:
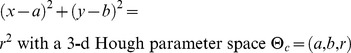
(2)The working principle for a classical HT is explained below.

**Figure 6:**
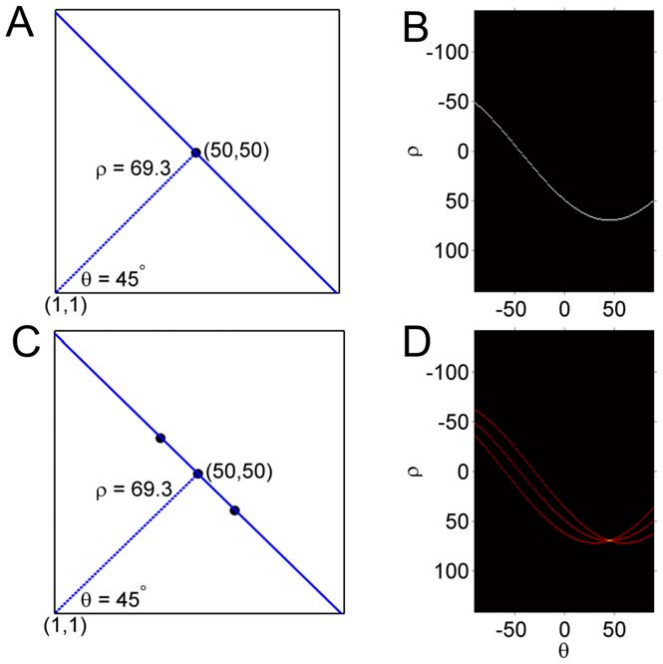
Illustration of working principle of the Hough Transform for lines. (A) Parametric normal form line passing through a point (50, 50) (B) Hough matrix parameter space with sinusoidal line corresponding to (50, 50). (C) 2 additional points added to (A). (D) Sinusoidal curves intersect for the three collinear points. One peak in the Hough space corresponds to one line in the image.

Fig. 6A represents the parametric normal form line 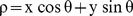, drawn in solid blue color, passing through a point (50, 50), with 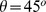 and 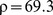. Here the origin is (1,1). Fig. 6B shows a sinusoidal curve in the Hough parameter space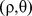, corresponding to the point (50,50) in the real space. When we have three points (Fig. 6C), the Hough parameter space has three sinusoidal curves (Fig. 6D) corresponding to the three points in real space and they have an intersection point corresponding to a particular pair of 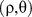 values indicating that the three points are collinear in the real space (Fig. 6C). The individual curves are accumulated in a matrix (the Hough matrix), and consensus lines are identifiable as peaks within this accumulation matrix (in this case, a single point with a value of 3). When there are multiple lines in the image space, there will be several intersections of the sinusoidal lines (peaks) for the group of points falling on the corresponding lines in the image space. Line end points are determined based on votes and a pre-defined maximum gap allowed between two points. If the distance between points exceeds a threshold the line is terminated at the previous point generating an end point.

**Figure 7:**
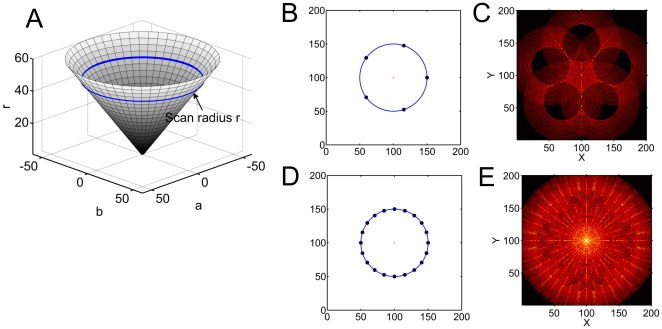
Illustration of working principle of the Hough Transform for circles. (A) Hough accumulator space for a circle *(a,b,r)* when the radius r is unknown. The scanning circles in the parameter space are on the cone surface in the 3-d space. (B) 5 points on a circle (100, 100, 50). (C) Circles in the Hough accumulator space corresponding to each of the input points in (B). (D) 20 points on a circle (100, 100, 50). (E) Circles in the Hough accumulator space corresponding to each of the input points in (D). The intersecting peak represents the center of the circle we are searching.

The detection of circles works on the same voting principle as that of lines, only the Hough parameter space is 3-d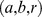. For each input point on the original circle (Fig. 7B) there will be a range of circles (depending on the discretization of the parameter space) in the Hough accumulator space with the input point as the center. The intersection of those circles will define the center of the circle in the original image space. For the above example with 5 and 20 points, the intersection of the circles in Hough space (Fig. 7C) is around (100,100) as the original circle (Fig. 7B). This example also shows how more input points, produces more votes for a particular circle increasing the probability of locating the center of the circles. The Hough space for multiple objects is shown in Figure S1. The accumulator slices are of the same size as the image space and the stack length is the total length of the radius range that has to be searched. So the accumulator array has a dimension of Image Width×Image Height×Length of Radius discretization. For objects with a known radius the search space is 2-d and calculations are much faster.

### Experiments

The detection of lines and circles using HT was performed for 100 random samplings of the data points on the structures at each data density. To remove spurious feature peaks in the Hough parameter space, we have used a 2-d median filter for lines and a discrete filter with a Laplacian of Gaussian kernel in order to smooth the 3-d Hough accumulator matrix for circles. To quantify the reconstruction, the structural similarity score was calculated for each random sample using a Complex Wavelet Structural Similarity Measure [49] (CW-SSIM) (Text S1) and the mean of those scores was calculated for each data density. These calculations were performed for different position noise and at different outlier noise densities as described above.

### Parameter Information for HT Reconstruction of Real Dataset

The parameter values are shown in Table 2 (a more detailed one is provided in Table S1). Here the Hough Matrix is denoted by H and cH for the lines and circles respectively. A 2-d median filtering was applied to H with sliding window = [length (row H); length (column H)]/75. A Laplacian of Gaussian filter (Text S2) and unsharp mask filter with parameter value of 0.2 was applied to the 3-d accumulation Hough matrix cH.

**Table 2:** HT Parameter information for the HT reconstruction of the real dataset.

Parameter	Value, Range
**Lines**	
*θ* (degree)	0.5, [−90, 89.5]
*ρ* (pixels)	6
maximum peaks	5000
peak separation (2 d) (pixels)	[15], [19] or [17], [19]
peak threshold	(0.23–0.26)×max(H)
minimum line length (pixels)	(126–168)
H bin gap filling (pixels)	(47.5–33.6)
**Circles**	
*r* (pixels)	0.4, [10, 120]×scale/pixelsize
maximum peaks	200
minimum spatial separation between peaks (pixels)	55
minimum radius separation between peaks (pixels)	(71–75)
peak threshold	(0.53–0.63)×max(cH)

[,] indicates fixed range values for all conditions and those in (-) are values that vary from 5–100% data density. The single values listed for the parameters *θ*, *ρ*, and *r* are the discretization steps. *scale = 25* and *pixelsize = 158 nm.* A detailed list of parameter values for all data densities (5% steps) are provided in the Table S1.

## Supporting Information

Text S1 **Structural Similarity Index Measure (SSIM).**
(DOCX)

Text S2 **Parameter Information for HT reconstruction of real dataset.**
(DOCX)

Figure S1 **Example of Hough space for multiple lines and circles in the real data (** **Fig. 5** **).** (A) Hough Matrix for the lines (microtubules) at 5% data density (B) Hough accumulator space for circles (CCPs) at 5% data density.
(TIF)

Figure S2 **Reconstruction measure using Structural Similarity Index CW-SSIM.** A total of 100 random simulations were performed at each data density and at outlier noise densities of 0 0.002, 0.005, 0.01, 0.02 and 0.05. Top row is for lines and bottom row is for circles Column (A) Position noise of 0. (B) Position noise of 5. (C) Position noise of 10.
(TIF)

Figure S3 **Crosstalk between red and green channel.** CCP(left) and Tubulin(right) data showing cross-talk from the green and red channel. Scalebar is 500 nm.
(TIF)

Figure S4 **Parallel line mask.**
(TIF)

Figure S5 **Laplacian of Gaussian (LoG) blob detection of circular features.** Multi-scale kernel size range is set to 1.0%–10% of the image size (1400×1400) and radius search range of 1.6–19 pixels which corresponds to ∼10 to 120 nm.It is a multiscale detection hence there are more than one circles with different radius for a detected blob. (A) Detection at 10% data density. (B) Same as (A), circles with radius less than 6 pixels (∼38 nm) are removed. (C) Close up view of the yellow region in (B). (D) Histogram of the detected bob radii in (B) (E) Detection at 50% data density. (F) Same as (E), circles with radius less than 6.5(∼41 nm) pixels are removed. (G) Close up view of the yellow region in (F). (H) Histogram of the detected bob radii in (F).
(TIF)

Table S1 **HT Parameter information for the HT reconstruction of the real dataset.** [,] indicates fixed range values for all conditions. The corresponding data density (%) is shown in brackets. The single values listed for the parameters *θ*, *ρ*, and *r* are the discretization steps. *Scale = 25* and *pixelsize = 158 nm.*
(DOCX)

Movie S1 HT reconstruction of lines shown in the mask (Fig. 1B) at position noise of 0 and noise densities of 0, 0.002, 0.005, 0.01, 0.02, 0.05.
(AVI)

Movie S2 HT reconstruction of lines shown in the mask (Fig. 1B) at position noise of 5 and noise densities of 0, 0.002, 0.005, 0.01, 0.02, 0.05.
(AVI)

Movie S3 HT reconstruction of lines shown in the mask (Fig. 1B) at position noise of 10 and noise densities of 0, 0.002, 0.005, 0.01, 0.02, 0.05.
(AVI)

Movie S4 HT reconstruction of circles shown in the mask (Fig. 1C) at position noise of 0 and noise densities of 0, 0.002, 0.005, 0.01, 0.02, 0.05.
(AVI)

Movie S5 HT reconstruction of circles shown in the mask (Fig. 1C) at position noise of 5 and noise densities of 0, 0.002, 0.005, 0.01, 0.02, 0.05.
(AVI)

Movie S6 HT reconstruction of circles shown in the mask (Fig. 1C) at position noise of 2 and noise densities of 0, 0.002, 0.005, 0.01, 0.02, 0.05.
(AVI)

Movie S7 HT reconstruction of parallel lines shown in the mask (Fig. S4) at position noise of 2 and noise densities of 0, 0.002, 0.005, 0.01, 0.02, 0.05.
(AVI)

Movie S8 HT reconstruction of the real data shown in (Fig. 5).
(AVI)
