# Supplementary material for: Inferring Biological Structures from Super-Resolution Single Molecule Images Using Generative Models
Source: PLoS One. 2012 May 22;7(5):e36973. doi: 10.1371/journal.pone.0036973 (PMC3358321; doi:10.1371/journal.pone.0036973)
Supplement: Text S1 — Structural Similarity Index Measure (SSIM). (DOCX) [file pone.0036973.s001.docx]

**Supporting Information Text S1. Structural Similarity Index Measure (SSIM)**

To measure the reconstructed structure with the original mask we have used a image similarity method called the Complex Wavelet Structural Similarity Index (CW-SSIM), which is an extension of spatial domain SSIM and inspired by the concept of the human visual system (HVS). It works on the principle that small geometric distortions produce a consistent phase shift in the local wavelet coefficients and that a consistent phase shift of the coefficients does not change the structural information content of the images. The advantage of this method is that it does not require explicit correspondences between pixels being compared and is insensitive to small geometric distortions hence it is robust to structural distortions, this approach may overestimate the similarity index by a small factor even if there is slight dissimilarity between the images being compared.

CW-SSIM Index is defined by:

$$\tilde{S}\left( c_{x},c_{y} \right)=\frac{2\left| \sum_{i=1}^{N} c_{x,i}c_{y,i}^{*} \right|+K}{{\sum_{i=1}^{N} \left| c_{x,i} \right|}^{2}+{\sum_{i=1}^{N} \left| c_{y,i} \right|}^{2}+K}$$

where $c_{x}= \left\{ c_{x,i} \right|i=1,...,N\}$ and $c_{y}=\{c_{y,i}|i=1,. ..,N\}$ are two sets wavelet coefficients obtained at the same spatial location in the same wavelet sub bands of the two images being compared and K is a small positive scalar constant which improves the robustness of CW-SSIM measure at low local signal to noise ratio. We have used this index for a simpler case since we compared binary images. We have tried to use other measures such as Multi-scale Structural SIM (MS-SIM), Information content Weighted SIM (IW-SSIM), and the standard SSIM, but on visual inspection they seem to underestimate the similarity score by a significant factor and we found CW-SSIM to be more appropriate.
