# Supplementary material for: Inferring Biological Structures from Super-Resolution Single Molecule Images Using Generative Models
Source: PLoS One. 2012 May 22;7(5):e36973. doi: 10.1371/journal.pone.0036973 (PMC3358321; doi:10.1371/journal.pone.0036973)
Supplement: Text S2 — Parameter Information for HT reconstruction of real dataset. (DOCX) [file pone.0036973.s002.docx]

**Supporting Information Text S2. Parameter Information for HT reconstruction of real dataset**

The discrete Laplacian of Gaussian (LoG) filter that we have used is given by:

$$LoG=\left[ \begin{matrix} 0 & 0 & 0 & -1 & -1 & -1 & 0 & 0 & 0 \\ 0 & -1 & -1 & -3 & -3 & -3 & -1 & -1 & 0 \\ 0 & -1 & -3 & -3 & -1 & -3 & -3 & -1 & 0 \\ -1 & -3 & -3 & 6 & 13 & 6 & -3 & -3 & -1 \\ -1 & -3 & -1 & 13 & 24 & 13 & -1 & -3 & -1 \\ -1 & -3 & -3 & 6 & 13 & 6 & -3 & -3 & -1 \\ 0 & -1 & -3 & -3 & -1 & -3 & -3 & -1 & 0 \\ 0 & -1 & -1 & -3 & -3 & -3 & -1 & -1 & 0 \\ 0 & 0 & 0 & -1 & -1 & -1 & 0 & 0 & 0 \end{matrix} \right]$$

The parameter values and ranges for each of the data densities (in steps of 5%) are shown in Table S1.
