# Supplementary figures and images for: Inferring Biological Structures from Super-Resolution Single Molecule Images Using Generative Models
Source: PLoS One. 2012 May 22;7(5):e36973. doi: 10.1371/journal.pone.0036973 (PMC3358321; doi:10.1371/journal.pone.0036973)

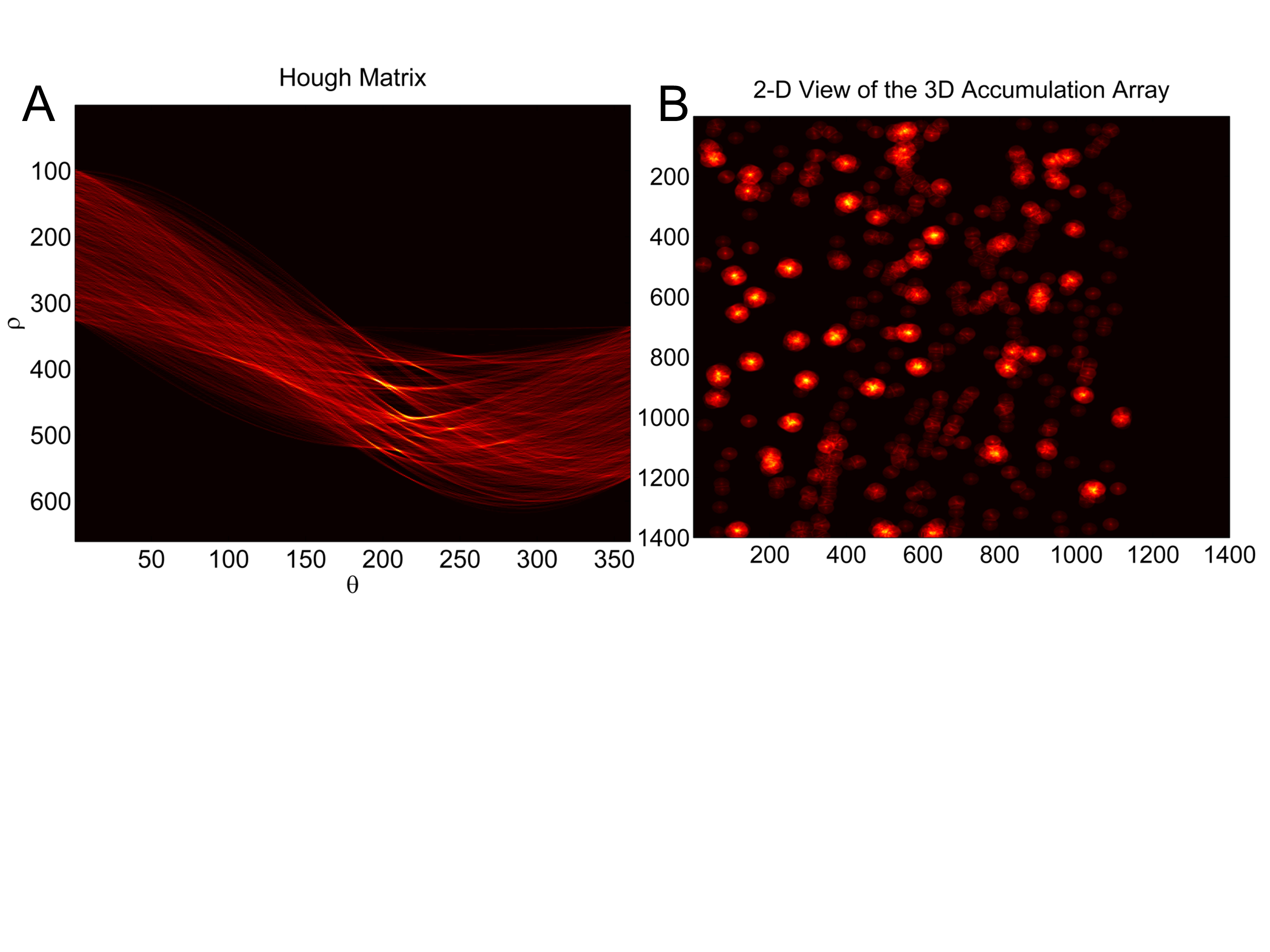

Supplement: Figure S1 — Example of Hough space for multiple lines and circles in the real data ( Fig. 5 ). (A) Hough Matrix for the lines (microtubules) at 5% data density (B) Hough accumulator space for circles (CCPs) at 5% data density. (TIF) [file pone.0036973.s003.tif]

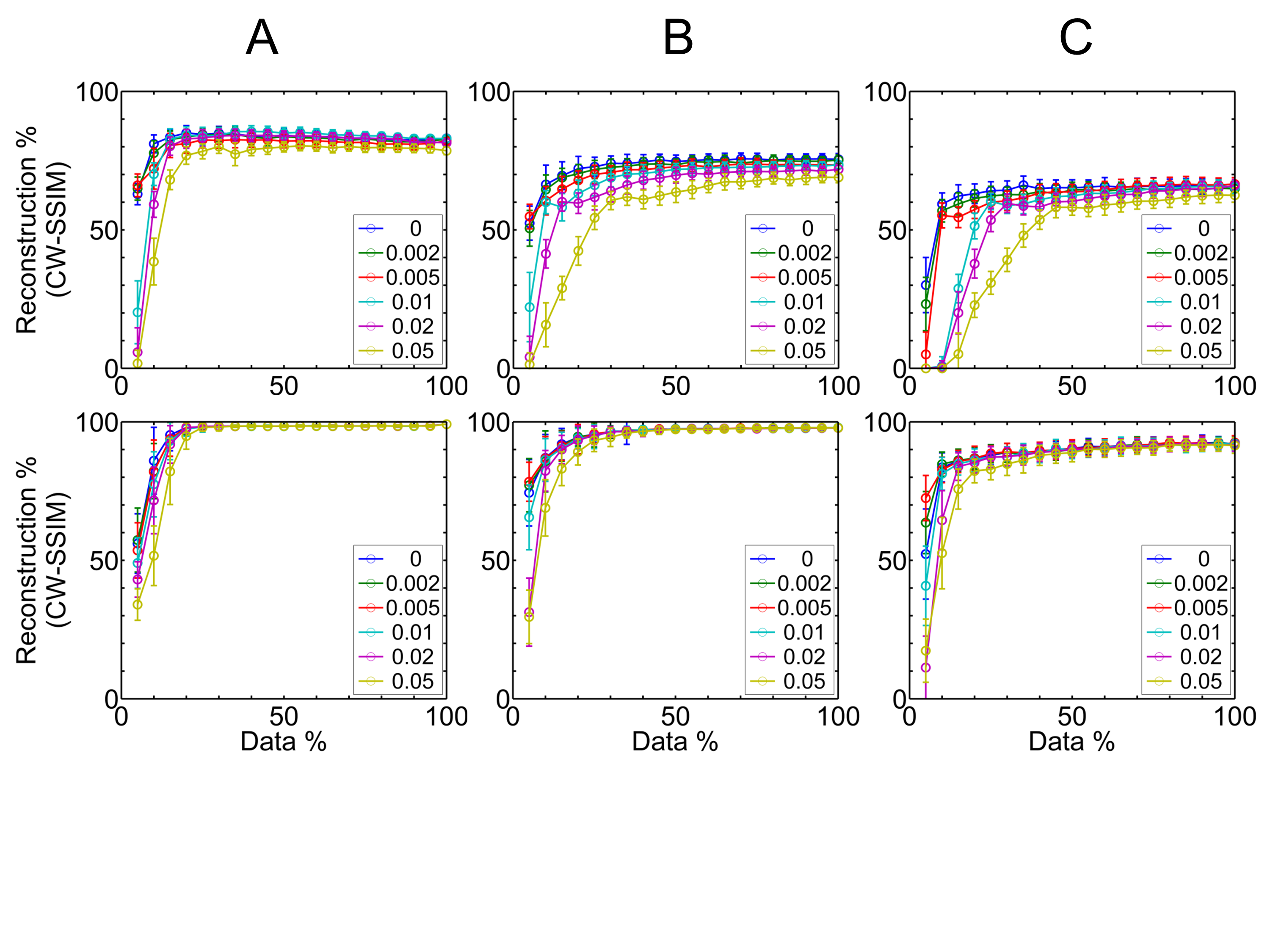

Supplement: Figure S2 — Reconstruction measure using Structural Similarity Index CW-SSIM. A total of 100 random simulations were performed at each data density and at outlier noise densities of 0 0.002, 0.005, 0.01, 0.02 and 0.05. Top row is for lines and bottom row is for circles Column (A) Position noise of 0. (B) Position noise of 5. (C) Position noise of 10. (TIF) [file pone.0036973.s004.tif]

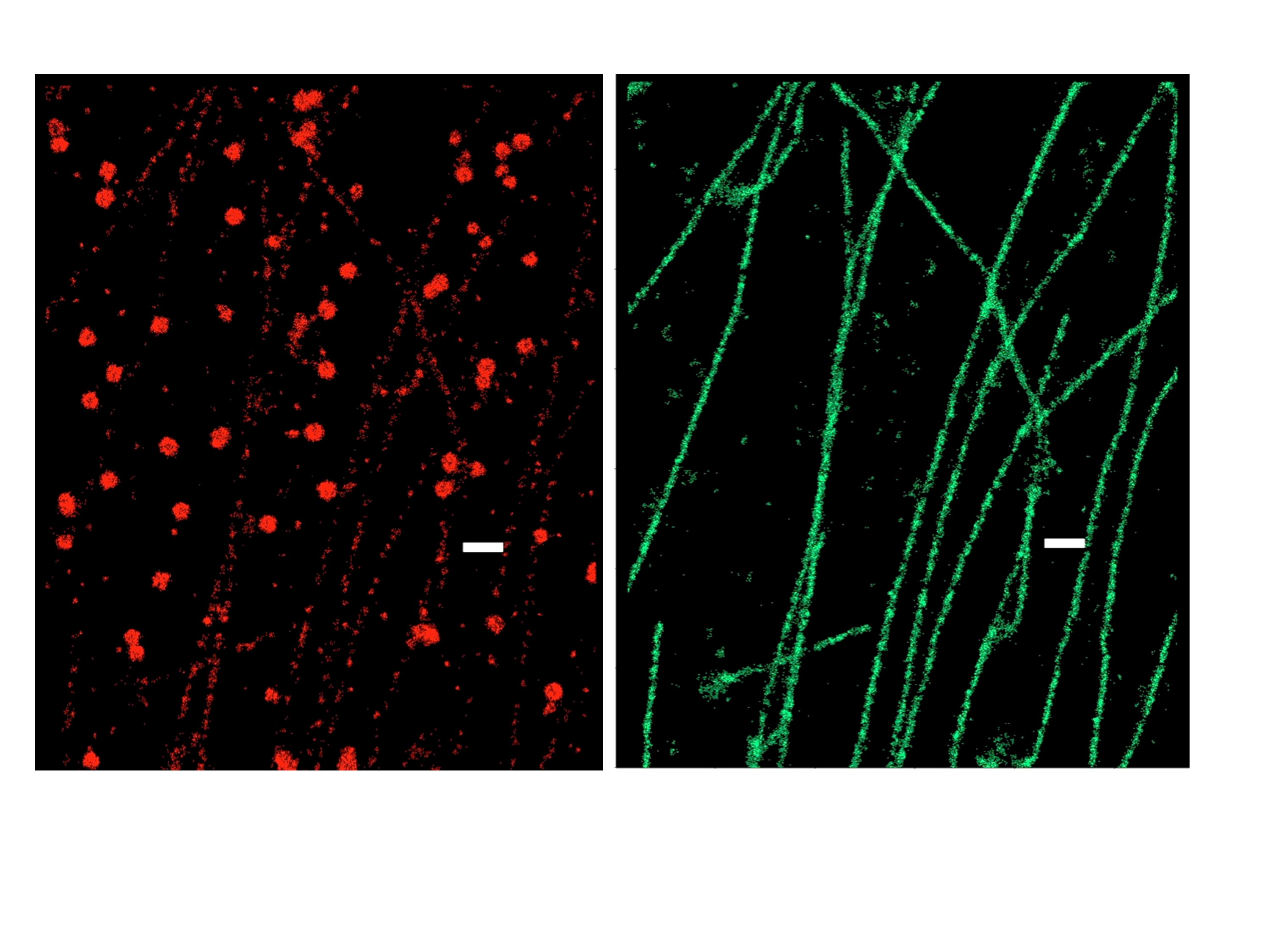

Supplement: Figure S3 — Crosstalk between red and green channel. CCP(left) and Tubulin(right) data showing cross-talk from the green and red channel. Scalebar is 500 nm. (TIF) [file pone.0036973.s005.tif]

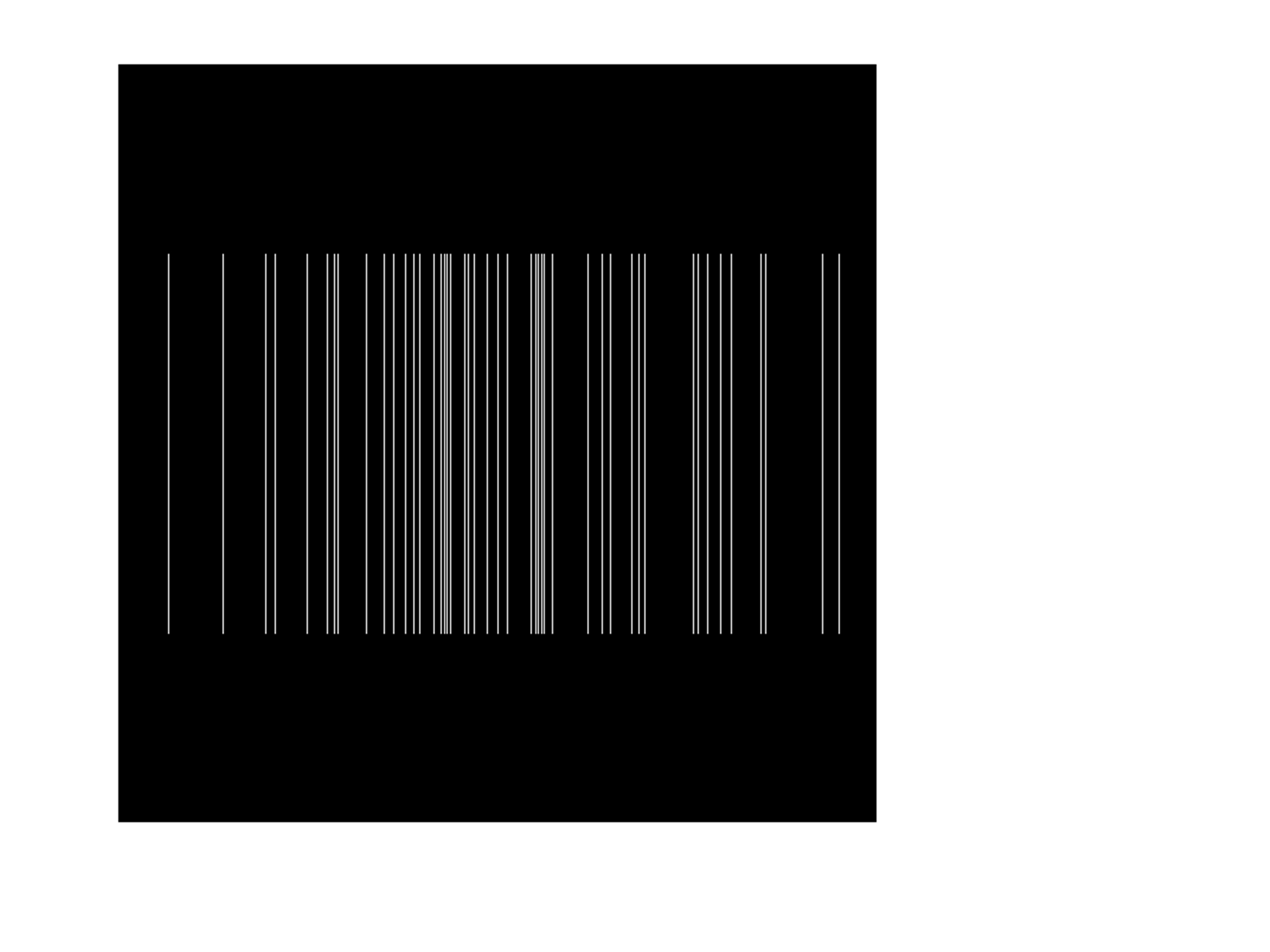

Supplement: Figure S4 — Parallel line mask. (TIF) [file pone.0036973.s006.tif]

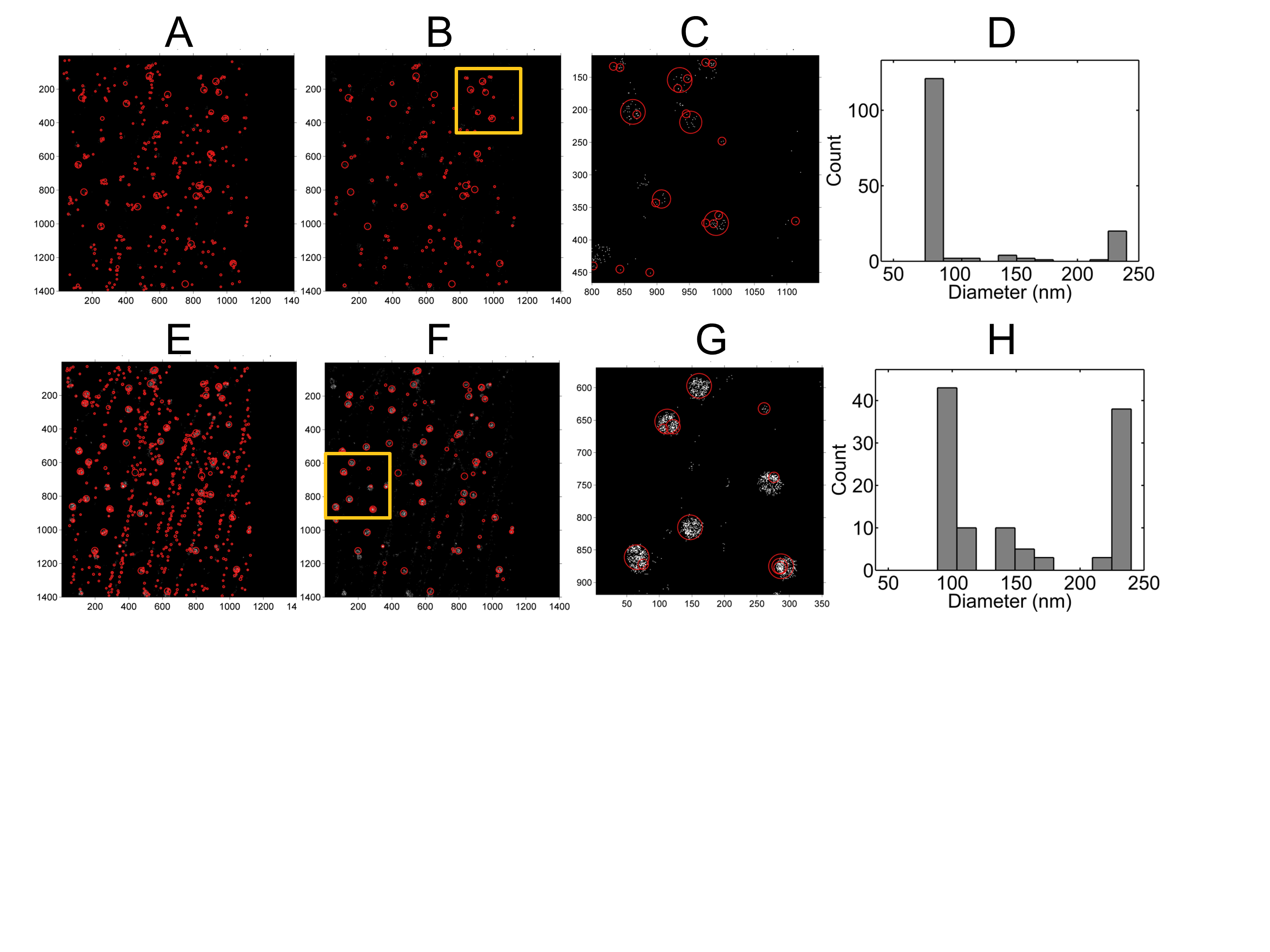

Supplement: Figure S5 — Laplacian of Gaussian (LoG) blob detection of circular features. Multi-scale kernel size range is set to 1.0%–10% of the image size (1400×1400) and radius search range of 1.6–19 pixels which corresponds to ∼10 to 120 nm.It is a multiscale detection hence there are more than one circles with different radius for a detected blob. (A) Detection at 10% data density. (B) Same as (A), circles with radius less than 6 pixels (∼38 nm) are removed. (C) Close up view of the yellow region in (B). (D) Histogram of the detected bob radii in (B) (E) Detection at 50% data density. (F) Same as (E), circles with radius less than 6.5(∼41 nm) pixels are removed. (G) Close up view of the yellow region in (F). (H) Histogram of the detected bob radii in (F). (TIF) [file pone.0036973.s007.tif]
