# Supplementary material for: Inferring Biological Structures from Super-Resolution Single Molecule Images Using Generative Models
Source: PLoS One. 2012 May 22;7(5):e36973. doi: 10.1371/journal.pone.0036973 (PMC3358321; doi:10.1371/journal.pone.0036973)
Supplement: Table S1 — HT Parameter information for the HT reconstruction of the real dataset. [,] indicates fixed range values for all conditions. The corresponding data density (%) is shown in brackets. The single values listed for the parameters θ, ρ, and r are the discretization steps. Scale = 25 and pixelsize = 158 nm. (DOCX) [file pone.0036973.s008.docx]

**Supporting Information Table S1. Parameter Information for HT reconstruction of real dataset**

| Parameter | Value, Range |
| --- | --- |
| **Lines** | |
| $\theta$ (degree) | 0.5, [-90, 89.5] |
| $\rho$(pixels) | 6 |
| maximum peaks | 5000 |
| peak separation (2d) (pixels) | [15, 19] (5 – 25 %, 65 – 100 %)  [17, 19] (30 – 60 %) |
| peak threshold | vf × max(H), vf =  0.229 (5 – 25 %)  0.228 (30 – 60 %)  0.261 (65 – 100 %) |
| minimum line length (pixels) | 126 (5 – 25 %)  168 (30 – 100 %) |
| H bin gap filling (pixels) | 47.5 (5 – 25 %)  33.6 (30 – 100 %) |
| **Circles** | |
| $r$ (pixels) | 0.4, [10, 120] × scale/pixelsize |
| maximum peaks | 200 (5 – 100 %) |
| minimum spatial separation between peaks (pixels) | 55 (5 – 100 %) |
| minimum radius separation between peaks (pixels) | 73 (5 – 15 %)  71 (20 – 60 %)  75 (65 – 100 %) |
| peak threshold | vf × max(cH), vf =  0.53 (5 – 15 %)  0.58 (20 – 40 %)  0.60 (45 – 60 %)  0.62 (65 – 80 %)  0.63 (85 – 100 %) |
